# Case Report: 1-Year Follow-Up of Vagus Nerve Stimulation in a Dog With Drug-Resistant Epilepsy

**DOI:** 10.3389/fvets.2021.708407

**Published:** 2021-07-20

**Authors:** Junya Hirashima, Miyoko Saito, Hirotaka Igarashi, Satoshi Takagi, Daisuke Hasegawa

**Affiliations:** ^1^Laboratory of Small Animal Surgery (Neurology), School of Veterinary Medicine, Azabu University, Sagamihara, Japan; ^2^Laboratory of Small Animal Internal Medicine, School of Veterinary Medicine, Azabu University, Sagamihara, Japan; ^3^Laboratory of Small Animal Surgery (Soft Tissue Surgery and Surgical Oncology), School of Veterinary Medicine, Azabu University, Sagamihara, Japan; ^4^Laboratory of Veterinary Radiology, Nippon Veterinary and Life Science University, Musashino, Japan; ^5^The Research Center for Animal Life Science, Nippon Veterinary and Life Science University, Musashino, Japan

**Keywords:** drug-resistant epilepsy, vagus nerve stimulation, dog, case report, neurostimulation, epilepsy surgery

## Abstract

A vagus nerve stimulation (VNS) system was surgically implanted to treat drug-resistant epilepsy in a 5-year-old male Shetland Sheepdog. At regular visits during a 1-year follow-up, treatment efficacy and adverse effects were assessed, and programmable stimulation parameters were adjusted to optimize stimulation intensity while avoiding adverse effects. The frequency of generalized tonic–clonic seizures was reduced by 87% after the initiation of VNS. The owner reported that the dog regained his personality, and the quality of life of both the dog and owner improved. The only adverse effect of VNS was a cough that was controlled by adjusting stimulation parameters. There were no surgical complications or other issues with the VNS device. This is the first long-term evaluation of VNS therapy in a dog, and the results obtained suggest that gradual adjustments of VNS parameters facilitate optimum VNS dosing.

## Introduction

Although epilepsy is a common neurological disorder in dogs, it is not controlled in approximately 30% of cases despite a correct diagnosis and appropriate medical treatment; and, thus, in those cases, it is called refractory epilepsy (1). Recently, the term drug-resistant epilepsy (DRE) has also been used. Surgery and neurostimulation therapy are clinically important treatment options for human epilepsy patients (1). Vagus nerve stimulation (VNS) is a type of neurostimulation that is becoming more widespread as adjunctive therapy for human epilepsy (2) because it reduces seizure frequency and improves quality of life (QOL) (2, 3).

In humans, the efficacy of VNS for epilepsy patients improves over time, with adjustments of stimulation parameters preventing adverse effects and increasing treatment efficacy. A long-term follow-up study on epilepsy patients for whom VNS parameters were gradually adjusted reported gradual reductions in median seizure frequency of 25, 40, and 53% after 3, 6, and 12 months of VNS, respectively (4). In veterinary medicine, two clinical studies on surgically implantable VNS and transcutaneous non-invasive VNS indicated the potential of VNS as adjunctive therapy for dogs with DRE (5, 6); mean reductions in seizure frequency of 34.4% and 25.9% were reported after 13 and 16 weeks of VNS, respectively. However, the long-term efficacy and safety of VNS in dogs and adjustments of VNS stimulus dosing remain unclear. A duration of at least 24 weeks has been proposed to assess the outcomes of therapeutic interventions for canine epilepsy (7). We herein describe the 1-year clinical course of VNS therapy in a dog with DRE, in which stimulus parameters were gradually adjusted during the year.

## Case Presentation

A 5-year-old non-castrated male Shetland Sheepdog with DRE was referred to Azabu University Veterinary Teaching Hospital (AUVTH) to evaluate the indication for VNS surgery. Frequent recurrent focal seizures (FS) and FS evolving into generalized tonic–clonic seizures (FS-GTCS) had occurred for 4 years. FS started with bilateral facial twitching or eyelid blinking, mastication, running without purpose, and immobilization and typically lasted for a few seconds to 1 min. FS evolving into GTCS started with the same motor activity before immediately developing into GTCS, which generally lasted 1–2 min. FS evolving into GTCS never continued for more than 5 min. The dog had been treated with various antiseizure drugs (ASDs), including zonisamide for 4 years, potassium bromide (KBr) for 3 years, phenobarbital for 2 years, levetiracetam for 6 months, and gabapentin for 5 months, with adequate dosing and serum concentrations (zonisamide, KBr, and phenobarbital) without good seizure control. The current ASD regimen consisted of zonisamide (8 mg/kg, BID, serum concentration: 56.8 μg/ml), gabapentin (15 mg/kg, TID), levetiracetam (40 mg/kg, TID), and KBr (20 mg/kg, BID, serum concentration: 1.2 mg/ml). Ursodeoxycholic acid and glycyrrhizinic acid were also administered due to slightly elevated liver enzymes. Despite strenuous attempts, seizure frequency progressively increased with countless FS every day and FS-GTCS clusters five to 14 times monthly for the last 6 months.

On presentation to AUVTH, the dog was alert and responsive, but restless. Physical and neurological examinations revealed no abnormalities. A complete blood count and serum biochemistry were unremarkable with the exception of a slight elevation of liver enzymes. X-ray showed microhepatica, and abdominal ultrasound revealed gallbladder mucocele. Fasting and postprandial total serum bile acid concentrations were 3.9 and 119 μmol/L, respectively. Computed tomography (CT) by the referring veterinarian showed no evidence of a portosystemic shunt or nodular lesions in the liver.

The Veterinary Medical Teaching Hospital of Nippon Veterinary and Life Science University diagnosed idiopathic epilepsy based on the Tier III level of the International Veterinary Epilepsy Task Force (IVETF) consensus proposal (8) 3 months prior to presentation to AUVTH. Magnetic resonance imaging (MRI) of the brain [3T, IVETF recommended protocol (9)] and a cerebrospinal fluid (CSF) analysis (proteins, cell counts, and cytology) revealed no abnormalities. Scalp electroencephalogram (EEG) under dexmedetomidine sedation was recorded using the recommended method (10). Despite semiology indicating FS, generally synchronized spike or spike-wave complexes were frequently observed in inter-ictal EEG (Supplementary Figure 1A). During EEG recordings, generalized electroencephalographic seizure activities without convulsions, that is, subclinical ictal EEG, were also noted (Supplementary Video). Based on EEG findings, the irritative zone remained unclear.

DRE was diagnosed based on these results and history, and liver cirrhosis was suspected to be caused by multiple ASD treatments, including phenobarbital. VNS was selected as adjunctive therapy because the epileptogenic zone was not identified, and less invasive surgery was preferable due to suspected cirrhosis. The owner also requested VNS therapy.

**Figure 1:**
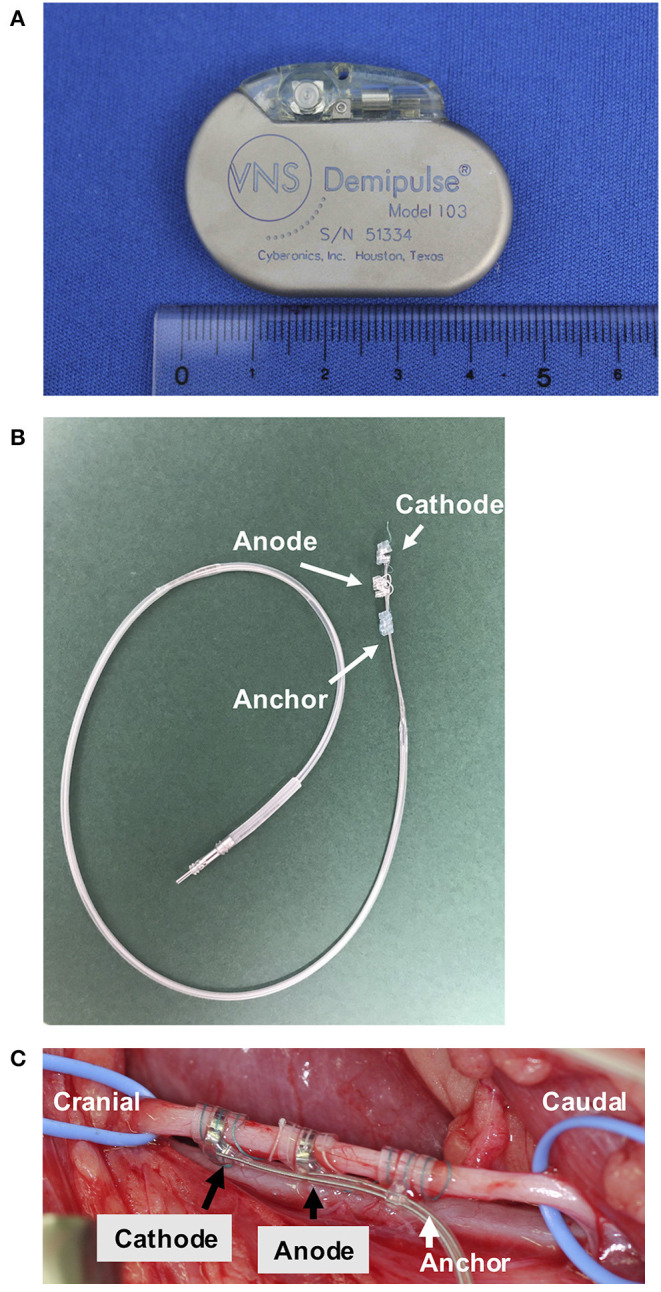
The VNS device and an intraoperative photograph of the left vagosympathetic trunk. The pulse generator (width 45 mm, height 32 mm, depth 6.9 mm, and weight 16 g) **(A)**. The electrode **(B)**. The left vagosympathetic trunk wrapped by the two helical electrodes and anchor tether **(C)**. VNS, vagus nerve stimulation.

A VNS device was implanted at AUVTH following ultrasound-guided Tru-Cut liver biopsy. The dog was premedicated with butorphanol and atropine. Anesthesia was induced with propofol and maintained with isoflurane. The VNS device comprised a pulse generator (Demipulse™ Model 103; LivaNova USA, Inc., Houston, TX, USA) and electrode lead with two helical electrodes at the tip (VNS lead M304, LivaNova USA, Inc., Houston, TX, USA) (Figures 1A,B). The method to implant the VNS device was previously described (5). Briefly, the two helical electrodes and anchor tether were wrapped around the left vagosympathetic trunk in the cervical area (Figure 1C), and the electrode lead was connected to the pulse generator implanted in the subcutaneous space cranial to the left scapula. Surgery was uneventful. After confirmation by system diagnostics of proper functioning, the device was maintained at 0 current.

A bandage for cast padding and a self-adherent bandage were applied around the neck to prevent postoperative seroma. During hospitalization, bile peritonitis occurred due to gallbladder rupture, and cholecystectomy was performed 6 days after VNS implantation. Recovery was uneventful. The liver and gallbladder were histologically diagnosed with chronic hepatitis progressing to cirrhosis and mucocele, respectively. The dog was hospitalized for 9 days. Ampicillin sodium/sulbactam sodium was intravenously administered during VNS surgery. Amoxicillin/clavulanate potassium was orally administered for 18 days after VNS implantation.

The device was activated after the neck wound had healed, in accordance with the manufacturer's instructions (11). The owner continued to record the number of FS, FS-GTCS, and any other seizures daily in the diary. The owner answered a visual analog scale (VAS) created for epilepsy surgery (Supplementary Data Sheet) in the day the dog was discharged. The VAS was completed again at the end of the 1-year follow-up. This survey was performed to investigate a subjective assessment of the owner to the treatment outcome of VNS therapy such as the QOL of both the dog and the owner.

Nine days after discharge, the dog presented to AUVTH for the first evaluation after VNS implantation. Physical and neurological examinations revealed no abnormalities. There was no seroma, and the wound had healed (Supplementary Figure 2A). The device was activated using an external programming system with stimulation parameters output current 0.25 mA, pulse width 250 μs, frequency 20 Hz, on-time 30 s, and off-time 5 min, which are initial settings commonly recommended for the treatment of epilepsy in humans (2, 11) (Supplementary Figure 3). There was no cough, voice change, bradycardia, or Horner's syndrome. The dog did not seem to take notice of the region around the left side of the neck where the vagosympathetic trunk was stimulated. The ASD regimen was unchanged from 3 months before VNS implantation to the 1-year follow-up.

Reevaluations were conducted approximately every 2 weeks in the initial post-implant period until the maximum tolerated stimulus current was reached and then every 4 weeks until the 1-year follow-up to screen for any adverse effects, check device function, and assess VNS efficacy. The output current was gradually increased by 0.25 in each visit based on tolerability and treatment efficacy (11). Other VNS parameters were adjusted to optimize and minimize adverse effects when required.

The owner was provided with a VNS external magnet, which may be used to stop seizures by swiping over the pulse generator to instantaneously provide an extra temporary stimulation. The output current of the magnet-induced stimulation (i.e., magnet mode) was configured at 0.25 mA above the cycling stimulation (i.e., normal mode). The other parameters of the magnet mode included a pulse width of 250 μs, frequency of 20 Hz, and on-time of 60 s.

In the second reevaluation after implantation (day 14 after VNS initiation), the general condition of the dog as well as seizure frequency and intensity remained unchanged. When the output current increased from 0.25 to 0.50 mA, the dog began to cough during a 30-s cycle of stimulation. Therefore, the pulse width was decreased to 130 μs, which prevented coughing while maintaining the output current.

**Figure 2:**
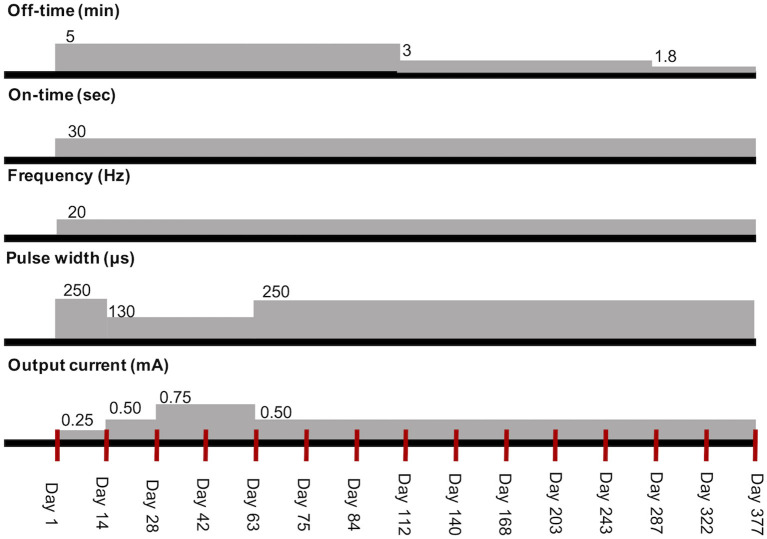
Transition of stimulation parameters of VNS therapy in the dog. The red vertical lines mean each visit. VNS, vagus nerve stimulation.

During the 1-year follow-up, stimulation parameters were adjusted in each visit based on the dog's responses (Figure 2). FS evolving into GTCS frequency began to decrease after day 14 but increased between days 42 and 63. There are strong fumes from exterior wall painting at a nearby apartment on days 53–62, and the dog became very nervous during this period. There was also a typhoon, which typically induced seizures in the dog, on days 46, 47, 54, and 55. During days 53–62, seizure frequency increased. A routine reevaluation was performed on day 63. It was then difficult to increase the output current without coughing. Therefore, to increase the stimulation intensity and not the output current, we increased the pulse width and decreased the output current while avoiding coughing.

After the output current was set to 0.5 mA and pulse width to 250 μs on day 63, it was not possible to increase the current to more than 0.5 mA without coughing. Since seizure frequency remained reduced and the owner was satisfied with the dog's condition, initial optimal dosing (i.e., highest efficient parameter setting without adverse effects) was attained on day 63, and a reevaluation was re-scheduled for every 4 weeks after day 84. The owner reported that the dog had regained his original character and slept well on day 112. The output current was not increased further due to coughing; therefore, the stimulation off-time was shortened at 3 and 1.8 min on days 112 and 287, respectively, according to a method described for humans (11, 12). On days 243 and 377, X-ray showed neither twisting of the electrode lead nor subcutaneous migration of the pulse generator (Supplementary Figure 4).

**Figure 3:**
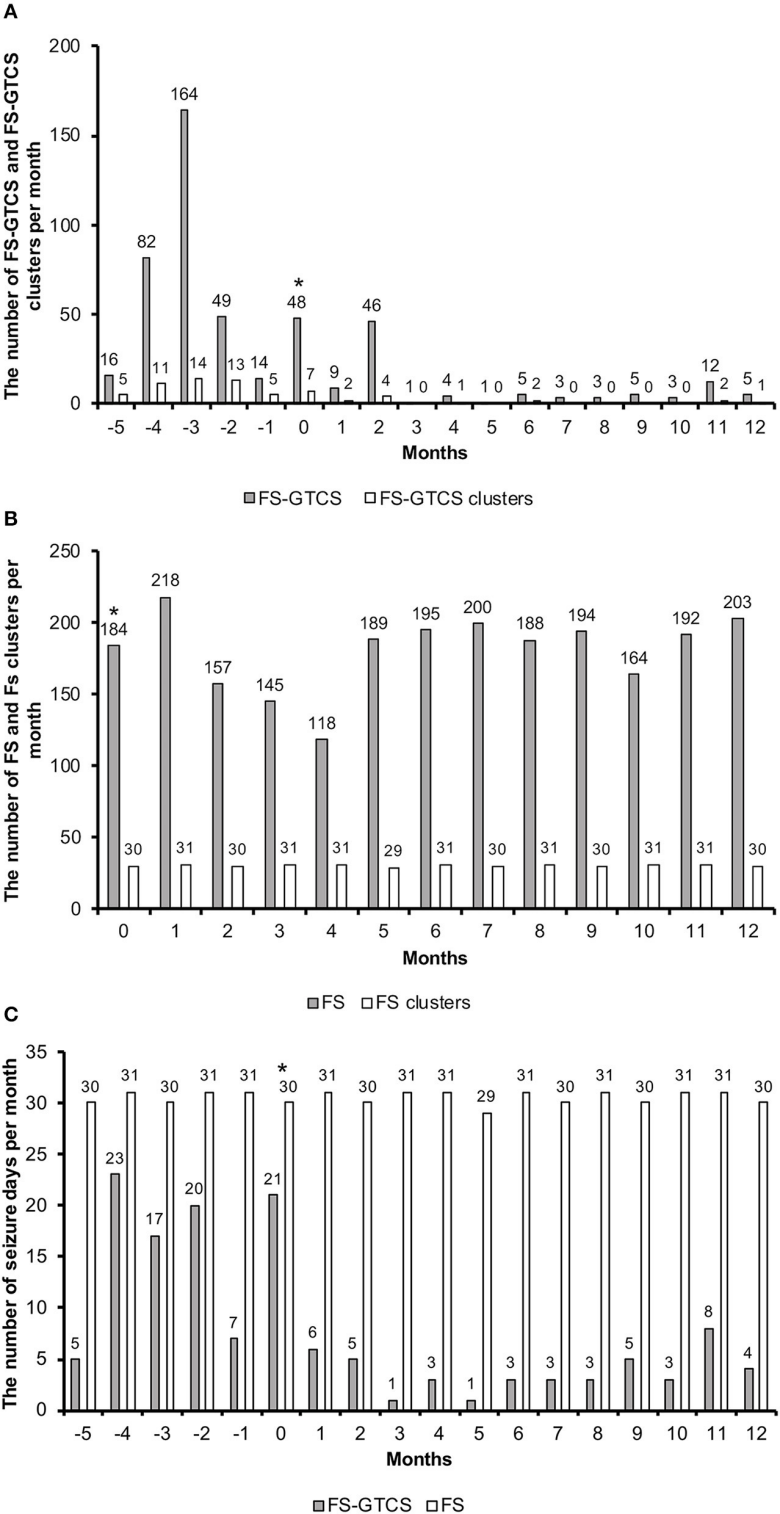
The number of FS-GTCS and FS-GTCS clusters per month **(A)**. The number of FS and FS clusters per month **(B)**. The number of seizure days (FS-GTCS and FS) per month **(C)**. The asterisk indicates that VNS therapy was initiated in this month. Months −5 to 0 indicate the retrospective period; months 1 to 12 indicate the follow-up period. FS-GTCS, focal seizures evolving into generalized tonic–clonic seizures; VNS, vagus nerve stimulation.

Throughout the 1-year follow-up, an 87% reduction was observed in the frequency of FS-GTCS (373 seizures for 6 months before VNS vs. 97 seizures in 1 year after VNS initiation). An 89% reduction in the frequency of FS-GTCS clusters was also achieved (55 clusters for 6 months vs. 12 clusters in 1 year) (Figure 3A). The number of FS-GTCS days also had 76% reduction (93 seizure days for 6 months vs. 45 seizure days in 1 year) (Figure 3C). Due to their very high frequency, the number of FS was not counted before VNS therapy, and, thus, there were no baseline data. The owner was instructed to count the number of FS after VNS initiation, and no decreases were noted during the 1-year follow-up (Figures 3B,C). However, VAS showed that the duration of FS shortened with measurement decreases from 100 to 15 mm. VAS also showed improvements in the QOL of the dog and owner with measurement decreases from 100 to 17 mm and from 93 to 13 mm, respectively (Table 1). Status epilepticus was not observed in the 6-month pretreatment or 1-year VNS treatment period.

**Table 1:** VAS scale before VNS initiation and at the end of the 1-year follow-up of VNS therapy.

**Questions**	**Before the initiation of VNS (mm)**	**At the end of the 1-year follow-up (mm)**
Frequency of focal seizures	100	70
Frequency of generalized seizures	100	57
Frequency of cluster seizures	100	54
Frequency of status epilepticus	0	0
Duration of focal seizures	100	15
Activity level of the dog or cat	100	41
Burden of medication on the dog or cat	100	81
Appetite of the dog and cat	100	55
Ataxia in the dog and cat	100	8
Consciousness level in the dog or cat	100	11
QOL of the dog or cat	100	17
QOL of the owner	92	13
Satisfaction with surgery		23.3
Frequency of seizure days		

*Details of VAS are shown in a supplementary data sheet. Measurements in millimeters are described in the table. Briefly, there are two descriptors representing extremes of answers for each question at the beginning and end (e.g., No FS and high frequency of FS). A longer measurement indicates a worse condition (a length of 100 mm is the worst). The question item of “Frequency of seizure days” was added in the VAS after the end of the 1-year follow-up (the supplementary data sheet used for this owner did not include this question). Thus, there were no answers from the owner. We determined to add this question because this information would help investigate the outcome of therapeutic investigations in canine epilepsy*.

*VNS, vagus nerve stimulation; QOL, quality of life; VAS, visual analog scale; FS, focal seizures*.

The owner used the handheld magnet at the onset of FS or the development of GTCS from FS. When the magnet was used during FS, subsequent GTCS did not occur; however, ongoing GTCS was not stopped. Once the duration of FS shortened, it was difficult to effectively use the magnet because FS had already stopped before the magnet was used.

The only adverse effect of VNS was a cough when the stimulation intensity increased. The owner reported that the dog tolerated VNS. There were no complications of surgery to implant the VNS device.

## Discussion

Adjustments of stimulation parameters to achieve optimum settings for each patient are important in VNS therapy. In humans, a higher stimulation intensity, particularly the output current, is generally more efficacious than a lower intensity (13, 14) but may not be necessary for some patients to achieve maximal anti-seizure effects, while a higher output current (>2.25 mA) may result in smaller reductions in seizure frequency (15). Parameter settings need to be adjusted to maximize efficacy and minimize complications. An initial low-intensity stimulus achieves better tolerance followed by gradual increases for accommodation to the stimulation (11, 13). In veterinary medicine, since there are currently no suggested methods for optimizing settings, we followed the initial settings recommended for the treatment of human patients and attempted incremental increases in each visit (2, 11, 13). Consequently, initial optimal dosing was successfully attained without persistent adverse effects within approximately 2 months in our dog.

As a tolerability strategy, if an increase in the output current is not tolerated, other stimulation parameters may be modified to accommodate tolerability. The dog began to cough after the initial increase in the output current to 0.5 mA. Therefore, the pulse width was reduced from 250 to 130 μs at the output current of 0.5 mA, which stopped the cough. After several weeks, the pulse width was reverted to 250 μs, and the cough was not elicited; therefore, the dog had habituated to the stimulation.

We shortened the stimulation off-time on days 112 and 287 to increase the duty cycle. A higher duty cycle increased VNS efficacy in some human patients (12). However, increasing the duty cycle was not beneficial in our dog (Figure 3). Further studies are needed to clarify the importance of the duty cycle in dogs.

It was difficult to increase the output current to more than 0.75 mA with a pulse width of 250 μs without coughing during the remaining treatment period. Consistent with the present results, a previous VNS study reported that it was not possible to increase the output current to more than 0.75 mA with a pulse width of 500 μs in nine out of 10 dogs without coughing (5). Despite a lower output current than that suggested for humans (2, 11), the anti-epileptic effects of VNS were confirmed in the dog of the present case report and the dogs reported by Muñana et al. (5). The vagus nerve comprises A-, B-, and C-fibers (16, 17). A study on rats showed that C-fibers were not associated with the anti-epileptic effects of VNS (18). A-fibers are myelinated somatic afferent and efferent fibers. B-fibers are myelinated efferent preganglionic autonomic fibers. A study on the activation threshold of each fiber type in anesthetized dogs reported that the thresholds of A- and B-fibers with a pulse width of 300 μs were 0.37 ± 0.18 and 1.6 ± 0.35 mA, respectively (16). The output current needed to treat our dog and the dogs reported by Muñana et al. (5) was markedly lower than the threshold of B-fibers. Although it currently remains unclear whether A- or B-fibers are more important for the anti-epileptic effects of VNS, even in humans, the outcomes of VNS in our dog and the dogs in the study by Muñana et al. (5) suggest that A-fibers contributed more to the anti-epileptic effects of VNS than B-fibers. Based on the present results and previous findings, an output current in the range of 0.25–0.75 mA is tolerated well by and effective for dogs and is the suggested setting for dogs with epilepsy.

VNS reduced not only seizure frequency but also seizure duration in humans (19). In the present study, the frequency of FS-GTCS decreased by 87% and that of FS-GTCS clusters by 89% in the 1-year treatment period. The number of FS-GTCS days also decreased by 76%. The duration of FS was also shortened with an 85% reduction in the VAS scale. These findings indicate that our dog achieved IVETF-defined partial therapeutic success for epilepsy (7).

The only adverse effect of VNS was coughing during the stimulation, which was successfully controlled by adjusting stimulation parameters. There were no surgical complications, such as a seroma at the incision site of the subcutaneous pocket for the pulse generator, which was a common complication in other VNS studies on dogs (5, 20). The surgical procedure in the study of Martlé et al. (20) reported the electrode lead twisting in 50% of the dogs. However, the VNS device operated well for 1 year with our surgical method that referred to the way reported in Muñana et al. (5). Therefore, the surgical procedure in the present and previous (5) studies and our postoperative treatment, that is, the neck bandage, may be preferable to prevent troubles of the electrode lead and a seroma.

There are several reasons why we selected non-pharmacological therapy for the dog in this case report. Few human patients control seizures with a third ASD when the first two ASD have failed (21), and, thus, non-pharmacological therapy is recommended for those cases. Although this has not yet been examined in the field of veterinary medicine, previous studies indicated many similarities in epilepsy between humans and dogs, such as its etiology and prevalence (22, 23). Therefore, from the negative result of humans (21) and similarities between humans and canine epilepsy, we determined increasing ASD dose was not beneficial for our dog. Moreover, in the present study, a hepatic disorder was suspected and chronic hepatitis was diagnosed. This hepatic problem also indicated increasing ASD was not appropriate. Therefore, we selected non-pharmacological therapy for the dog.

Among non-pharmacological therapies, VNS was selected for the dog in this case report for a number of reasons. The epileptogenic zone was hard to detect on preoperative examinations (i.e., ictal and interictal EEG, MRI, and seizure semiology). Human candidates for VNS are patients who are not appropriate for epilepsy surgery with craniotomy due to, for example, an unclear epileptogenic zone or a high risk of complications (2). The epileptogenic zone of the dog was not identified, and the suspected hepatic disorder indicated that less invasive surgery was preferable. Moreover, VNS is effective for a wide range of patients regardless of the seizure type (2) and was beneficial for the dog, which had several seizure types.

Previous VNS clinical trials in veterinary medicine did not use the VNS external magnet (5, 6). The termination of seizure activity and shortening of seizure durations and severity using a magnet-induced stimulation have been reported in humans (24). The owner used the magnet at the onset of FS activity to stop the development of GTCS. This immediate effect is an additional benefit of VNS therapy. Therefore, further studies are preferable to investigate the effects of the VNS external magnet in dogs.

The VAS showed considerable improvements in the QOL of both the dog and owner, with high satisfaction with VNS by the owner (Table 1). The QOL of the dog improved with VNS therapy based on the restoration of his gentle character, improved sleep quality, and enjoyment of walking with other dogs. The QOL of the owner also improved because the dog looked happy and she also began to sleep well. Although the dog is not free of seizures, VNS therapy is an effective adjunctive treatment for this dog because of the markedly reduced frequency of FS-GTCS and improved QOL.

Our dog in this case report was nice to handle in the clinic. However, even if a dog is not so fond of clinics, it does not become a reason for excluding VNS therapy from treatment options because the stimulation parameters could be adjusted easily and quickly just by externally holding the programing wand over the dog's shoulder where the generator is located.

We reevaluated the dog every 2 weeks until the stimulation parameters were optimized for the dog. Such frequent visits could be difficult for some owners. We anticipate that a monthly visit is also appropriate, although more frequent visits during the titration phase may allow for faster achievement to the optimal stimulus setting.

To the best of our knowledge, this is the first long-term evaluation of VNS therapy in which stimulus parameters were adjusted during follow-ups to optimize VNS dosing in the dog. The present results suggest the potential benefits of VNS as adjunctive non-pharmacological therapy and the benefit of gradual adjustments of stimulation parameters while avoiding adverse effects in dogs with DRE. The suggested settings and protocol for adjustments of parameters in dogs need to be verified in further studies.

## Data Availability Statement

The original contributions presented in the study are included in the article/Supplementary Material, further inquiries can be directed to the corresponding author/s.

## Ethics Statement

The animal study was reviewed and approved by the Ethics Committee of Azabu University, Japan. Written informed consent was obtained from the owners for the participation of their animals in this study.

## Author Contributions

JH drafted the article, contributed to the neurological management of the patient, and acquired data. MS conceived the case report, performed VNS surgery, contributed to the neurological diagnosis and management of the patient, and critically revised the manuscript. DH diagnosed the dog with DRE and critically revised the manuscript. HI performed liver biopsy, contributed to the hepatic management of the patient, and critically revised the article. ST performed cholecystectomy and critically revised the article. All authors contributed to editing the manuscript and approved the submitted version.

## Conflict of Interest

The authors declare that the research was conducted in the absence of any commercial or financial relationships that could be construed as a potential conflict of interest.
