# Supplementary material for: Case Report: 1-Year Follow-Up of Vagus Nerve Stimulation in a Dog With Drug-Resistant Epilepsy
Source: Front Vet Sci. 2021 Jul 20;8:708407. doi: 10.3389/fvets.2021.708407 (PMC8330973; doi:10.3389/fvets.2021.708407)
Supplement: Supplementary file 1 [file Data_Sheet_1.PDF]

Visual Analog Score for the owner of an animal undergoing epilepsy surgery

Date: mm/dd/yy (preoperative•postoperative day•mth•year)

Owner's name: ______________________ Name of the dog/cat: ______________________

Please mark with a vertical line the point on the horizontal line that most accurately describes your feelings or impression to each question.

1. Frequency of focal seizures

None ⟵――――――――――――――――――⟶ High ________ mm

2. Frequency of generalized seizures

None ⟵――――――――――――――――――⟶ High ________ mm

3. Frequency of cluster seizures

None ⟵――――――――――――――――――⟶ High ________ mm

4. Frequency of status epilepticus

None ⟵――――――――――――――――――⟶ High ________ mm

5. Duration of focal seizures

Short ⟵――――――――――――――――――⟶ Long ________ mm

6. Activity level of the dog or cat

High* Low

←——————————————→ ________ mm

*Same as before the onset of epilepsy

7. Burden of medication on the dog or cat

No drugs Burdensome

←——————————————→ ________ mm

8. Appetite of the dog or cat

Normal None

←——————————————→ ________ mm

9. Ataxia in the dog or cat

None Severe*

←——————————————→ ________ mm

*Hard to walk or stand up

10. Consciousness level in the dog or cat

Normal Severe*

←——————————————→ ________ mm

*Always sleeping

11. Quality of life of the dog or cat

Good Bad*

______ mm

*Considering euthanasia

12. Quality of life of the owner

Good Bad

______ mm

13. Satisfaction with surgery

Good* Bad*

______ mm

*Good: Glad my pet underwent surgery

*Bad: Regret subjecting my pet to surgery
