# Supplementary material for: Case Report: 1-Year Follow-Up of Vagus Nerve Stimulation in a Dog With Drug-Resistant Epilepsy
Source: Front Vet Sci. 2021 Jul 20;8:708407. doi: 10.3389/fvets.2021.708407 (PMC8330973; doi:10.3389/fvets.2021.708407)
Supplement: Supplementary file 2 [file Data_Sheet_2.PDF]

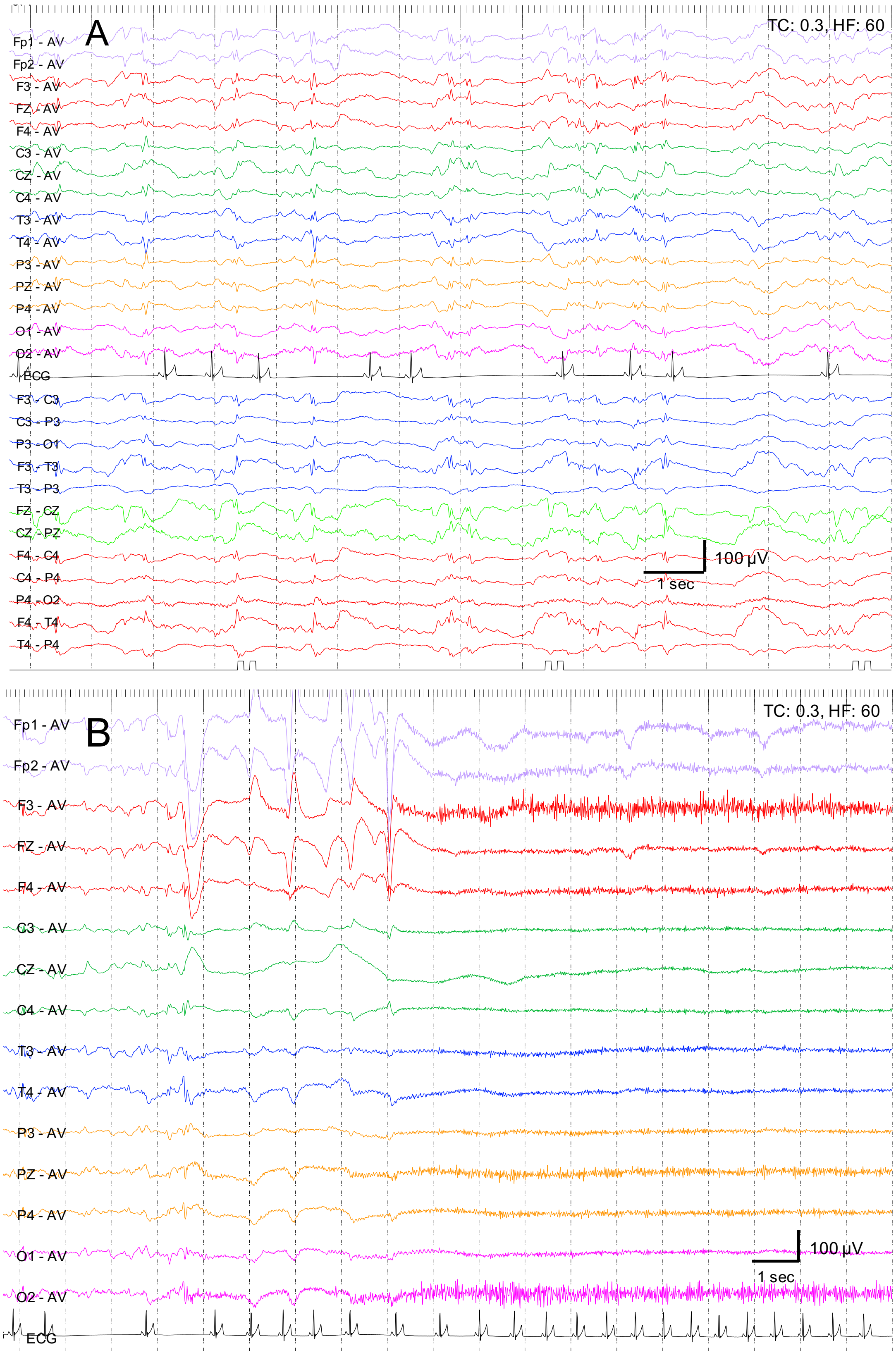
A
TC: 0.3, HF: 60
Fp1 - AV
Fp2 - AV
F3 - AV
FZ - AV
F4 - AV
C3 - AV
CZ - AV
C4 - AV
T3 - AV
T4 - AV
P3 - AV
PZ - AV
P4 - AV
O1 - AV
O2 - AV
ECG
F3 - C3
C3 - P3
P3 - O1
F3 - T3
T3 - P3
FZ - CZ
CZ - PZ
F4 - C4
C4 - P4
P4 - O2
F4 - T4
T4 - P4
100 µV
1 sec
B
TC: 0.3, HF: 60
Fp1 - AV
Fp2 - AV
F3 - AV
FZ - AV
F4 - AV
C3 - AV
CZ - AV
C4 - AV
T3 - AV
T4 - AV
P3 - AV
PZ - AV
P4 - AV
O1 - AV
O2 - AV
ECG
100 µV
1 sec

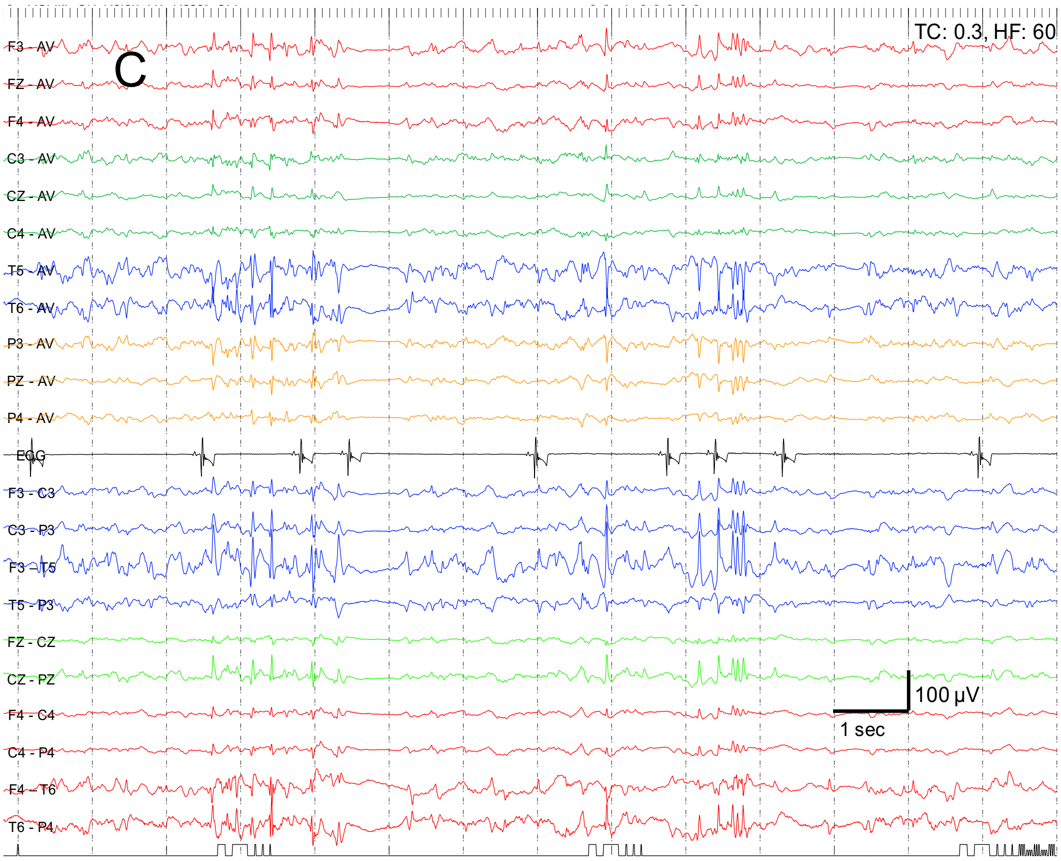
TC: 0.3, HF: 60
C
F3 - AV
FZ - AV
F4 - AV
C3 - AV
CZ - AV
C4 - AV
T5 - AV
T6 - AV
P3 - AV
PZ - AV
P4 - AV
ECG
F3 - C3
C3 - P3
F3 – T5
T5 - P3
FZ - CZ
CZ - PZ
F4 - C4
C4 - P4
F4 – T6
T6 - P4
100 µV
1 sec

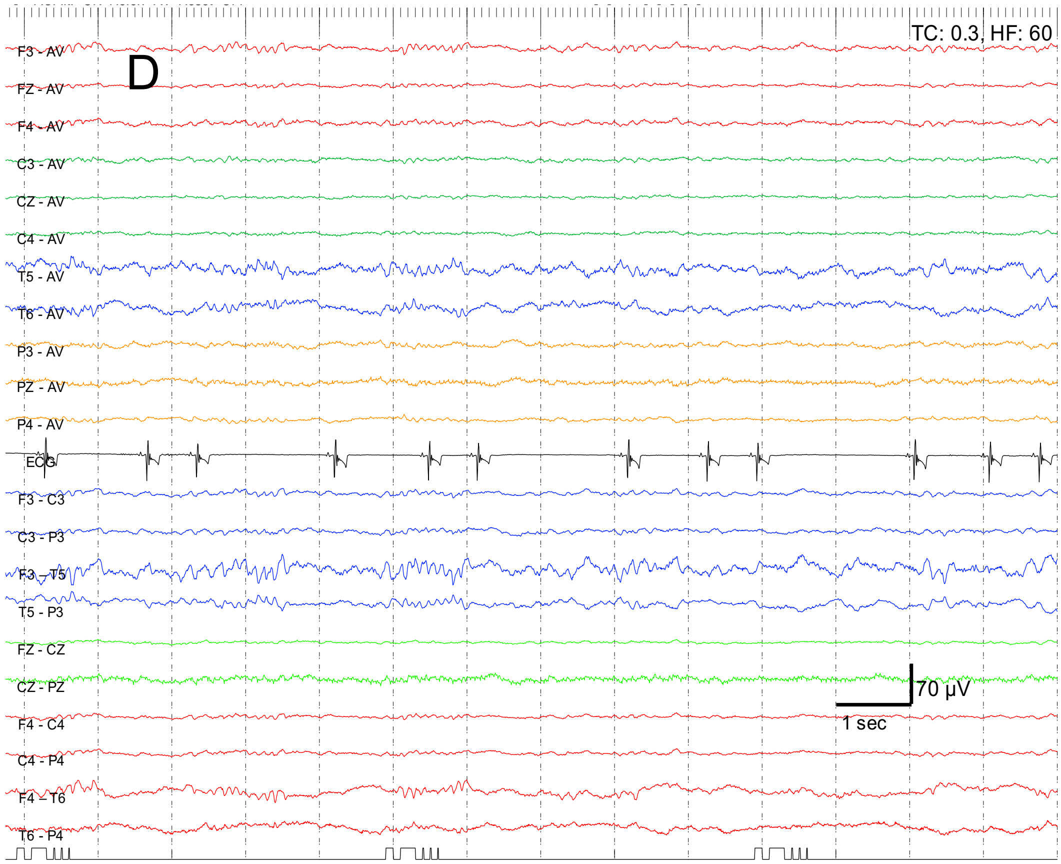
TC: 0.3, HF: 60
D
F3 - AV
FZ - AV
F4 - AV
C3 - AV
CZ - AV
C4 - AV
T5 - AV
T6 - AV
P3 - AV
PZ - AV
P4 - AV
ECG
F3 - C3
C3 - P3
F3 – T5
T5 - P3
FZ - CZ
CZ - PZ
F4 - C4
C4 - P4
F4 – T6
T6 - P4
70 µV
1 sec

Supplementary Figure 1. Scalp electroencephalogram (EEG) of the dog before the initiation of VNS at the Veterinary Medical Teaching Hospital of Nippon Veterinary and Life Science University (A, B) under sedation with dexmedetomidine and 11 months after the initiation of VNS at Azabu University Veterinary Teaching Hospital (C, D) under sedation with medetomidine. Some differences were observed in the montage between hospitals. Generally synchronized spikes or spike-wave complexes were frequently found in inter-ictal EEG (A). These interictal epileptiform discharges (IEDs) were detected in most EEG traces. Electroencephalographic seizure activity, which was obtained during inter-ictal EEG measurements, showed a generalized low voltage and very fast activity (B). An increased heart rate also indicated that this EEG activity was an electroencephalographic seizure. The dog started blinking and pricked up his ears without convulsion during generalized seizure activity, which lasted for 12 seconds (see also Supplementary movie). Although inter-ictal EEG 11 months after the initiation of VNS still frequently showed IEDs (C), EEG traces had no IEDs around the end of EEG recording, during which time the effects of medetomidine began to diminish (D).
