# Supplementary material for: Case Report: 1-Year Follow-Up of Vagus Nerve Stimulation in a Dog With Drug-Resistant Epilepsy
Source: Front Vet Sci. 2021 Jul 20;8:708407. doi: 10.3389/fvets.2021.708407 (PMC8330973; doi:10.3389/fvets.2021.708407)
Supplement: Supplementary file 3 [file Data_Sheet_3.PDF]

frontiers

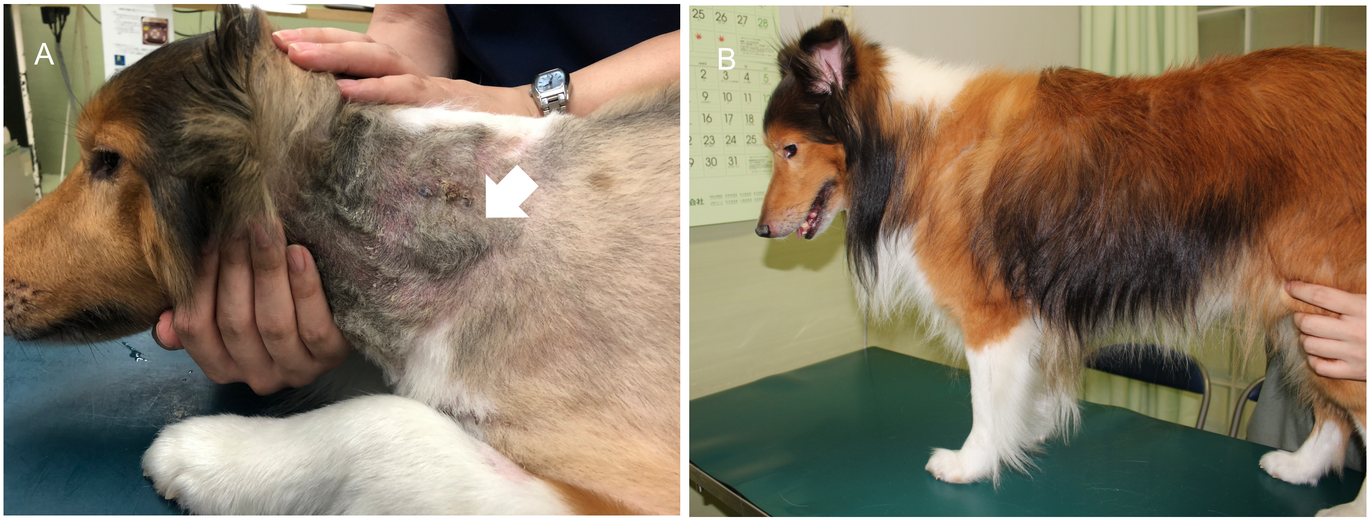

Supplementary Figure 2. Appearance of the dog on day 1 (18 days after VNS surgery) (A) and day 377 (B). There was no seroma or other issues with the wound. The white arrow shows the pulse generator under the skin (A). Once hair grew back, the pulse generator was no longer noticeable (B).
