# Supplementary material for: Case Report: 1-Year Follow-Up of Vagus Nerve Stimulation in a Dog With Drug-Resistant Epilepsy
Source: Front Vet Sci. 2021 Jul 20;8:708407. doi: 10.3389/fvets.2021.708407 (PMC8330973; doi:10.3389/fvets.2021.708407)
Supplement: Supplementary file 4 [file Data_Sheet_4.PDF]

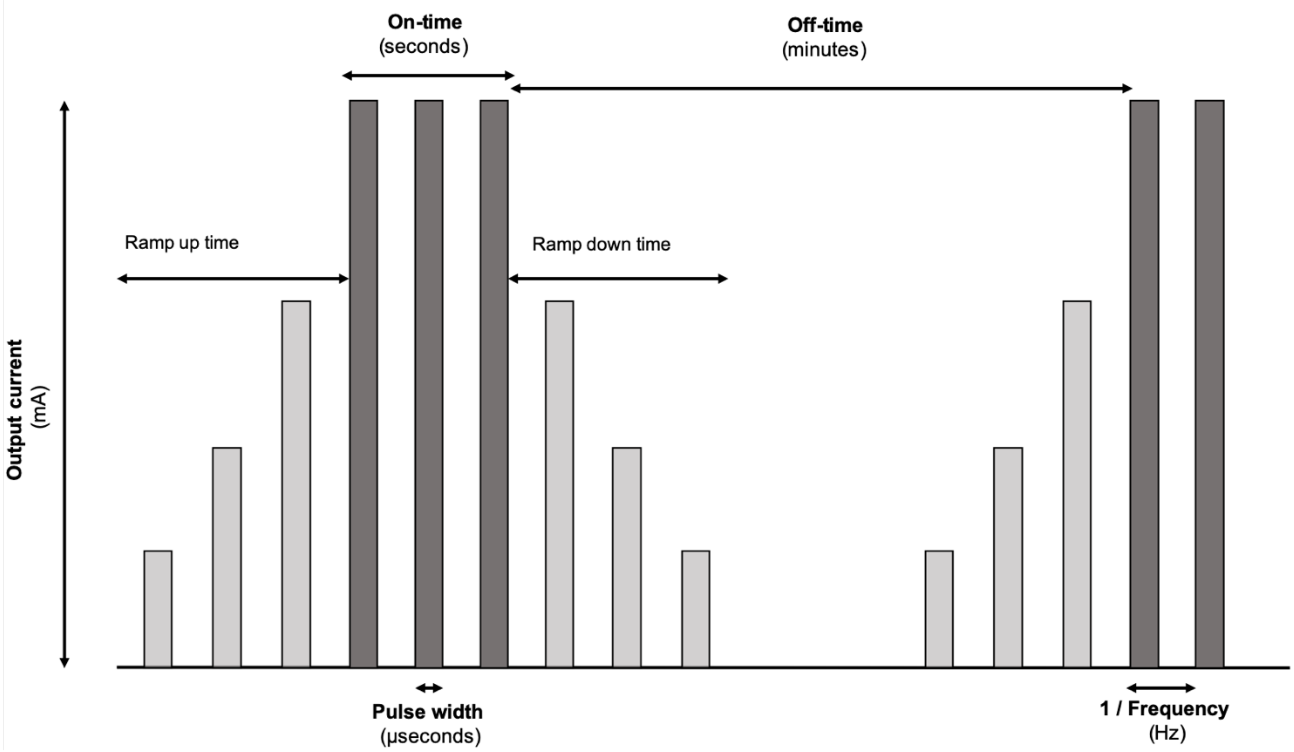

Supplementary Figure 3. Stimulation parameters of the vagus nerve stimulation. Output current, pulse width, frequency, on-time, and off-time were adjustable using an external programming system.
