# Supplementary material for: Case Report: 1-Year Follow-Up of Vagus Nerve Stimulation in a Dog With Drug-Resistant Epilepsy
Source: Front Vet Sci. 2021 Jul 20;8:708407. doi: 10.3389/fvets.2021.708407 (PMC8330973; doi:10.3389/fvets.2021.708407)
Supplement: Supplementary file 5 [file Image_4.pdf]

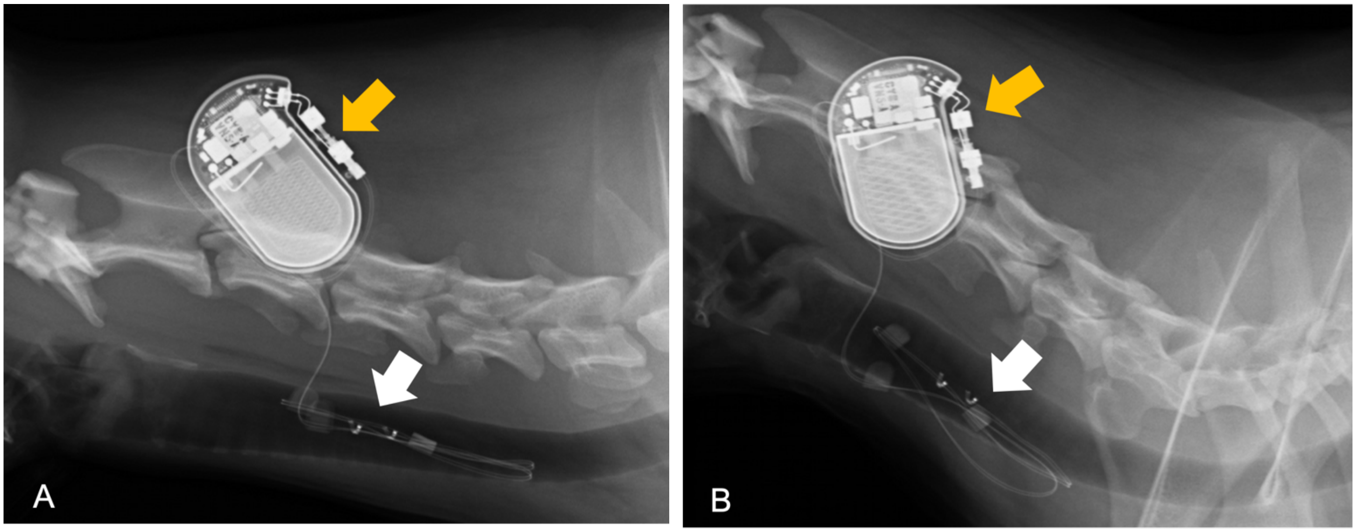

Supplementary Figure 4. Radiographs of the neck on days243 (A) and 377 (B).Neither twisting of the electrode lead (white arrow) nor subcutaneous migration of the pulse generator (yellow arrow) was observed.
